# Astragaloside IV attenuates ferroptosis-related testicular injury in rats with experimental varicocele

**DOI:** 10.3389/fphar.2026.1893309

**Published:** 2026-10-07

**Authors:** Boxian Gao, Shengjing Liu, Mengxin Sun, Weiyi Chen, Zhuozhuo Shi, Jixiang Gao, Chongfu Zhong

**Affiliations:** 1 Shandong University of Traditional Chinese Medicine, Jinan, China; 2 China Academy of Chinese Medical Sciences Xiyuan Hospital, Beijing, China; 3 Shandong First Medical University, Jinan, China; 4 Affiliated Hospital of Shandong University of Traditional Chinese Medicine, Jinan, China

**Keywords:** AR, astragaloside IV, ferroptosis, MBOAT2, varicocele

## Abstract

Varicocele is an important correctable cause of male infertility. Oxidative stress and lipid peroxidation contribute to the associated testicular injury, but the role of ferroptosis remains controversial. Astragaloside IV (AS-IV), a quality-control metabolite of Astragali radix, has antioxidant activity and can modulate ferroptosis-related processes, yet its effect in varicocele is unclear. This study evaluated whether AS-IV attenuates testicular injury in experimental varicocele and examined ferroptosis-related mechanisms. A Sprague–Dawley rat model was established by partial ligation of the left renal vein and treated with oral AS-IV; Ferrostatin-1 served as a pharmacological comparator. Label-free proteomics, histopathology, biochemical assays and targeted molecular analyses were combined with a TM4 Sertoli-cell model of RSL3-induced injury. AS-IV lessened tubular injury and improved Johnsen scores in a dose-related manner, reduced testicular Fe^2+^, ROS and MDA, and raised the GSH/GSSG ratio. Proteomics showed partial reversal of lipid-metabolism and ferroptosis-related enrichment after AS-IV. These changes were accompanied by recovery of androgen receptor (AR) and MBOAT2. In TM4 cells, AS-IV improved viability and lowered C11-BODIPY oxidation after RSL3; enzalutamide weakened the effects of AS-IV on lipid peroxidation and MBOAT2 and only partly reduced the viability rescue. The findings provide experimental evidence that AS-IV ameliorates varicocele-related testicular injury and that AR/MBOAT2-related signalling may participate.

## 1 Introduction

Infertility affects approximately one in six people of reproductive age worldwide, and male factors account for about half of cases (Liu et al., 2024). Varicocele (VC) is of major clinical importance in male infertility. Its prevalence may reach 80% among men with secondary infertility, and it is a common correctable cause of male infertility (Crafa et al., 2024). The pathology of VC mainly involves impaired local microcirculation and venous reflux, which can raise testicular temperature and produce hypoxia, oxidative stress and accumulation of metabolites, thereby affecting testosterone production, the blood–testis barrier (BTB) and spermatogenesis (Kang et al., 2022).

Microsurgical spermatic-vein ligation is a commonly used treatment and can improve semen parameters and pregnancy outcomes in some patients, but a subset of patients do not obtain a satisfactory fertility benefit (Çayan et al., 2025; Szabó et al., 2025). These differences indicate that current treatment does not resolve the problem in all patients and that adjunctive or non-surgical strategies for selected populations remain worth exploring (Minhas et al., 2021; Bento et al., 2025).

Oxidative stress is considered an important pathological component of VC-related testicular injury (Finelli et al., 2022). Increased reactive oxygen species (ROS), imbalance of the antioxidant system and elevated lipid-peroxidation products have been observed in patient samples and animal models (Barati et al., 2020; Lv et al., 2022). Iron can promote oxidative injury through the Fenton reaction (Yuan et al., 2023). Ferroptosis, a regulated cell-death programme driven by iron-dependent lipid peroxidation, has therefore attracted increasing attention (Mishima et al., 2025).

Although ferroptosis has been linked to various male reproductive disorders, its specific role in VC-related testicular injury remains controversial. Ferroptosis-related changes have been detected in semen from men with VC, but animal studies have not consistently observed alterations of the canonical GPX4 pathway in testicular tissue (Sun et al., 2023; Shaygannia et al., 2021; Mo et al., 2025; Moretti et al., 2024).

Ferroptosis is governed by a complex regulatory network. Recent studies in AR-positive tumour cells have shown that the androgen receptor (AR) can transcriptionally upregulate membrane-bound O-acyltransferase 2 (MBOAT2), thereby altering phospholipid fatty-acyl composition, reducing selected polyunsaturated fatty acid (PUFA) peroxidation substrates and constituting an anti-ferroptotic mechanism distinct from GPX4 and FSP1 (Li et al., 2023). Decreased AR expression has been reported in testicular tissue from patients with VC and from animal models (Soares et al., 2013; Zalata et al., 2013; Guido et al., 2014). Given the high hormonal sensitivity of the testis, the AR/MBOAT2 axis may participate in the regulation of lipid peroxidation under stress.

Astragali radix (Huangqi) is the dried root of *Astragalus membranaceus* (Fisch.) Bunge or *Astragalus membranaceus* var. *mongholicus* (Bunge) P.K.Hsiao and has long been used as a tonic botanical drug in China and neighbouring regions. Its recorded medicinal use dates to the Shennong Bencao Jing of the Han dynasty, where it was listed among superior-grade drugs; the Mingyi Bielu describes it as tonifying depletion in men. The current Pharmacopoeia of the People’s Republic of China monographs these botanical sources and lists the cycloartane triterpenoid saponin astragaloside IV (AS-IV) as one of the quality-control markers. AS-IV is therefore both a characteristic metabolite of Astragali radix and a chemically defined entity linking traditional medicinal use to modern pharmacological evaluation. In traditional medical terms, varicocele is often grouped with “sinew tumours” as a disorder of local vessel stasis; the pathogenesis is commonly ascribed to qi stagnation and blood stasis. Qi is regarded as the commander of blood, and deficient qi may fail to move blood and thereby aggravate local stasis. Astragali radix is used to tonify qi, and its traditional applications include assisting blood movement in qi-deficiency-related stasis. Modern studies have shown that aqueous extracts of Astragali radix can improve human sperm motility, and that AS-IV can upregulate AR and modulate ferroptosis-related processes in models of reproductive toxicity (Sirasanagandla et al., 2025) and other oxidative injury (Li et al., 2024a; Shi et al., 2025; Wang et al., 2022a). On the basis of this traditional-use background and a reproducible chemical identity, the present study evaluated commercially supplied, purity-defined AS-IV in a rat varicocele model and examined whether ferroptosis-related processes and AR-associated signalling participate in its effects.

On the basis of the above evidence, we hypothesised that VC may be accompanied by reduced testicular AR expression and downregulation of MBOAT2, thereby disturbing lipid homeostasis and increasing cellular susceptibility to ferroptosis, and that AS-IV may restore AR/MBOAT2-related signalling, improve lipid and redox status and thereby attenuate testicular injury. To test this hypothesis, a VC rat model was used, with Ferrostatin-1 (Fer-1) as a pharmacological positive control, together with multi-dose AS-IV intervention, proteomics, histopathology, biochemical assays and targeted molecular measurements.

## 2 Materials and methods

### 2.1 Materials and instruments

*In vivo* experiments used astragaloside IV (AS-IV; CAS 84687-43-4; molecular formula C41H68O14; purity 98%; Energy Chemical, China; structure shown in Figure 1a). Cell experiments used a second preparation of AS-IV (catalogue HY-N0431; CAS 84687-43-4; purity 99.93% by HPLC; MedChemExpress). The two preparations share the same chemical identity and were used for gavage and cell treatment, respectively. Ferrostatin-1 (Fer-1; catalogue S7243; purity 99.98%; Selleck Chemicals LLC, United States), RSL3 (catalogue CM00895; CAS 1219810-16-8; Proteintech, Wuhan Sanying Biotechnology Co., Ltd.) and enzalutamide (MDV3100; catalogue S1250; CAS 915087-33-1; Selleck Chemicals) were also used. The mouse Sertoli cell line TM4 (CL-0456) and its matching complete medium (CM-0456) were purchased from Procell Life Science and Technology Co., Ltd. (Wuhan, China).

**FIGURE 1:**
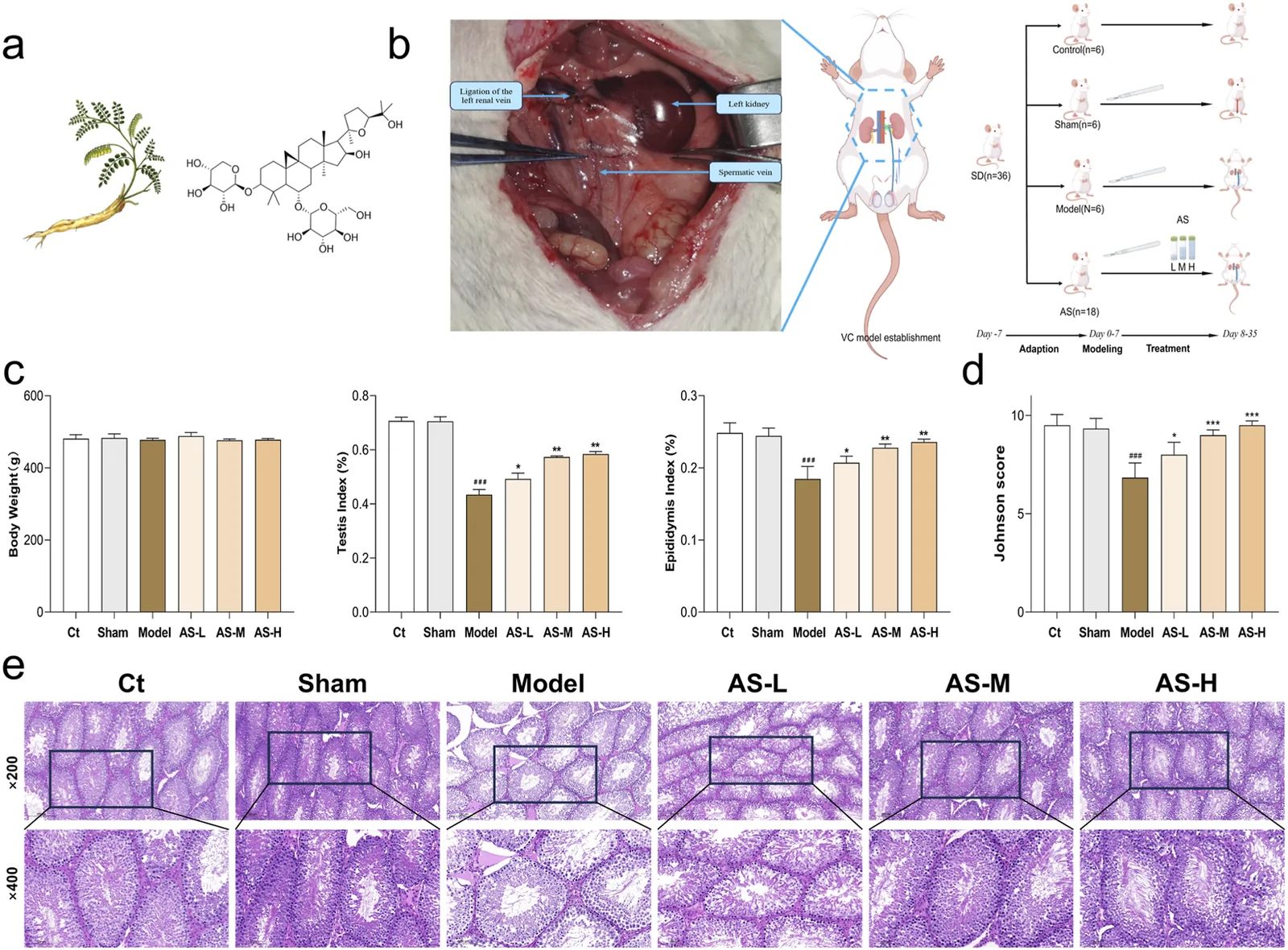
AS-IV attenuates testicular histological injury in VC model rats. **(a)** Molecular structure and source of AS-IV. **(b)** Partial ligation of the left renal vein to establish the VC model and the grouping and intervention schedule. **(c)** Terminal body weight and testicular and epididymal organ indices. **(d,e)** Johnsen score and H&E staining. Scale bars: as labelled in figure (200 μm). Bars show mean ± SD; n = 6 animals. Compared with the blank control group: ###*P* < 0.001; compared with the model group: *P < 0.05, **P < 0.01, ***P < 0.001.

Assay kits were purchased from Servicebio Technology Co., Ltd. (Wuhan, China) and included a ferrous iron assay kit (G4323), a total glutathione/oxidized glutathione assay kit (G4304), a malondialdehyde (MDA) assay kit (G4302), a tissue ROS assay kit (DHE fluorescence, G1746), an RNA extraction reagent (G3013) and a chloroform substitute (G3014). H&E staining was performed with a kit from Nanjing Jiancheng Bioengineering Institute (D026-1). Cell viability was measured with Cell Counting Kit-8 (catalogue KGA9305-500; KeyGEN BioTECH, Jiangsu). Membrane lipid peroxidation was measured with a lipid peroxidation assay kit (BODIPY 581/591 C11; catalogue S0043S; Beyotime Biotechnology, China).

Primary antibodies included rabbit anti-AR (22089-1-AP; Proteintech, Wuhan, China), rabbit anti-MBOAT2 (bs-18709R; Bioss, Beijing, China), rabbit anti-β-actin (MD6553; MDL, China) and rabbit anti-ZO-1 (21773-1-AP; Proteintech, Wuhan, China). All primers were synthesised by Servicebio Technology Co., Ltd.

Major instruments included a chemiluminescence imaging system (ChemiScope 6100; Clinx, China), a real-time PCR system (CFX Connect; Bio-Rad, United States), a flow cytometer (NovoCyte 2060R; ACEA Biosciences/Agilent, United States), an automated microplate reader (WD-2120B; Beijing LIUYI Biotechnology Co., Ltd.), an inverted fluorescence microscope (MF53; Guangzhou Micro-shot Optical Instrument Co., Ltd.), a transmission electron microscope (HT7800; Hitachi, Japan) and a scanning electron microscope (Quanta 250; FEI, United States).

### 2.2 Animals and ethics statement

Seventy-two 8-week-old specific-pathogen-free male Sprague–Dawley rats weighing 240 ± 20 g were purchased from Beijing Vital River Laboratory Animal Technology Co., Ltd. [licence No. SCXK (Jing) 2021-0011]. Animals were housed at the Animal Experimental Centre of the Affiliated Hospital of Shandong University of Traditional Chinese Medicine, six per cage, at 22 °C ± 1 °C and 60% ± 2% relative humidity, under a 12 h/12 h light–dark cycle, with free access to standard chow and water. Animals were acclimatised for 1 week before experiments.

All animal experiments followed the Guidelines for Ethical Review of Laboratory Animal Welfare and were approved by the Animal Ethics Committee of the Affiliated Hospital of Shandong University of Traditional Chinese Medicine (approval No. 2021-40).

### 2.3 Establishment of the VC model, grouping and intervention

As shown in Figure 1b, experimental VC was established by partial ligation of the left renal vein using the Turner method. Rats were anaesthetised with 3% pentobarbital sodium (40 mg/kg, intraperitoneal injection), prepared and placed in the supine position. A midline abdominal incision was made to expose the left kidney and left renal vein. A metal probe 0.85 mm in diameter was placed beneath the left renal vein, which was then partly ligated with 4-0 silk while blood flow was preserved. After confirmation of no obvious renal ischaemia within 2 min, the abdomen was closed layer by layer. In the sham-operated group the left renal vein was isolated but not ligated. Clear tortuosity and dilatation of the left spermatic vein were used as the criterion of successful modelling. Previous studies have shown that this method can induce testicular injury and impaired spermatogenesis in rats at 7–28 days after surgery (Lv et al., 2022; Antonuccio et al., 2020; Jia et al., 2024).

In the first cohort, rats were assigned by a random-number table to six groups (n = 6 per group): blank control, sham operation, model (VC plus vehicle), and low-dose (20 mg/kg), mid-dose (40 mg/kg) and high-dose (80 mg/kg) AS-IV. The second cohort was likewise randomised to six groups (n = 6 per group): sham operation, model, low-, mid- and high-dose AS-IV, and a ferroptosis-inhibitor group (VC plus Fer-1 1 mg/kg, intraperitoneal injection) (Zhao et al., 2020; Liu et al., 2020; Yang et al., 2014).

AS-IV was dissolved in 0.5% sodium carboxymethyl cellulose (CMC-Na) and administered by daily gavage for 4 consecutive weeks. Fer-1 was first dissolved in DMSO and then diluted with saline. Body weight and general condition were monitored daily. At the end of the intervention, rats were killed under deep anaesthesia and testes were rapidly collected for subsequent analyses.

### 2.4 TM4 cell culture and drug treatment

The mouse Sertoli cell line TM4 was cultured in the dedicated complete medium at 37 °C in a humidified incubator with 5% CO2. The medium was replaced two to three times per week. Cells were passaged at a ratio of 1:3 when confluence reached approximately 80%–90%. Cells in logarithmic growth and of good appearance were used for experiments. On the basis of preliminary experiments, working concentrations were set at AS-IV 5 μM, enzalutamide 10 μM and RSL3 1.5 μM. Each drug was diluted to the working concentration in complete medium, and the final solvent concentration was kept consistent across groups.

The formal experiment comprised five groups: Control, RSL3, RSL3 + AS-IV, RSL3 + enzalutamide, and RSL3 + AS-IV + enzalutamide. In the corresponding groups, 5 μM AS-IV and/or 10 μM enzalutamide were added first and incubated for 24 h; 1.5 μM RSL3 was then added for a further 24 h while the original concentrations of AS-IV and enzalutamide were maintained. Thus the total treatment time was 48 h for AS-IV and enzalutamide and the last 24 h for RSL3. The RSL3 group received an equal volume of solvent for the first 24 h and then 1.5 μM RSL3 for 24 h. The Control group received an equal volume of solvent throughout. After the intervention, cell viability, lipid peroxidation, immunoblotting and cellular immunofluorescence were assessed.

### 2.5 Haematoxylin and eosin (H&E) staining

Testicular tissue was fixed in 4% paraformaldehyde for 24 h, embedded in paraffin and cut into 4 μm sections. H&E staining was performed by conventional methods and examined under a light microscope. Two discontinuous mid-testicular sections were examined per animal. Spermatogenesis was scored on the 10-point Johnsen scale. Only approximately circular cross-sections of seminiferous tubules were included. At least 30 eligible tubules were scored per animal, and the mean score was taken as the individual value for that animal. Slides were recoded before evaluation and scored by an observer unaware of group assignment.

### 2.6. Scanning electron microscopy (SEM)

Testicular tissue was fixed in 2.5% glutaraldehyde, dehydrated through graded ethanol, critical-point dried and sputter-coated with gold. The surface and architecture of seminiferous tubules were examined by scanning electron microscopy.

### 2.7 Transmission electron microscopy (TEM)

Testicular tissue was fixed in 2.5% glutaraldehyde, rinsed with 0.1 mol/L phosphate buffer (pH 7.4), post-fixed in 1% osmium tetroxide for 2 h, dehydrated through graded ethanol and embedded in resin. Ultrathin sections of 50–70 nm were prepared and examined by transmission electron microscopy for cellular integrity and mitochondrial morphology.

### 2.8 Proteomic analysis

Rat testicular tissue stored at −80 °C was placed in ice-cold lysis buffer, homogenised by sonication and centrifuged at 12,000 × g for 15 min at 4 °C. The supernatant was collected and protein concentration was determined by the BCA method. Protein was reduced with dithiothreitol, alkylated with iodoacetamide in the dark, precipitated with cold acetone at −20 °C and digested overnight at 37 °C with sequencing-grade trypsin at an enzyme-to-protein ratio of 1:50. Peptides were desalted on C18 spin columns, dried under vacuum and analysed by nanoLC–MS/MS on a Thermo Scientific Q Exactive HF-X in data-dependent acquisition mode. Peptides were separated on an Acclaim PepMap C18 column (75 μm × 250 mm) over a 120-min gradient. Raw spectra were searched with MaxQuant v1.6.2.10 against the UniProtKB rat reference proteome (UP000002494). Quantification used MaxLFQ (minimum ratio count = 1; match between runs). The false-discovery rate for protein and peptide identification was 1%. Subsequent statistics were performed in Perseus v1.5.1.6.

### 2.9 Immunofluorescence

Paraffin sections of testis were dewaxed and rehydrated, and antigen retrieval was performed in sodium citrate buffer. After cooling and blocking, sections were incubated overnight at 4 °C with anti-ZO-1 primary antibody and then with a fluorescent secondary antibody for 50 min at room temperature. Nuclei were counterstained with DAPI, slides were mounted, and images were acquired under a fluorescence microscope.

TM4 cells treated as described in Section 2.4 were rinsed with PBS and fixed in 4% paraformaldehyde for 15 min at room temperature. After PBS washes, cells were blocked with 5% BSA at 37 °C for 30 min. The blocking solution was removed without washing, and cells were incubated overnight at 4 °C with ZO-1 primary antibody (1:200). After warming, cells were incubated with a fluorescent secondary antibody in the dark at room temperature, nuclei were counterstained with DAPI, slides were mounted, and images were acquired under a fluorescence microscope with identical exposure parameters across groups.

### 2.10 Measurement of lipid-peroxidation-related indices and Fe^2+^ in testicular tissue

Testicular homogenates were prepared and supernatants collected. ROS, MDA, the GSH/GSSG ratio and Fe^2+^ content were measured according to the kit instructions.

### 2.11 Western blot analysis

Testicular tissue was homogenised in RIPA lysis buffer containing protease inhibitors. After centrifugation, protein concentration was determined by the BCA method. Equal amounts of protein were denatured, separated by SDS-PAGE and transferred. After blocking, membranes were incubated overnight at 4 °C with primary antibodies against AR (1:5000), MBOAT2 (1:1000) and β-actin (1:1000), washed and incubated with HRP-conjugated secondary antibody (1:5000) for 1.5 h. Bands were visualised by ECL and quantified with ImageJ.

Cell samples followed the same immunoblotting procedure, with differences only in sample preparation and antibody conditions. After treatment as described in Section 2.4, TM4 cells were scraped in RIPA lysis buffer containing protease inhibitors, disrupted and centrifuged. The supernatant was collected and protein concentration was determined by the BCA method. Equal amounts of protein were denatured, separated by SDS-PAGE and transferred. After blocking, membranes were incubated overnight at 4 °C with anti-MBOAT2 (1:1000), washed and incubated with HRP-conjugated goat anti-rabbit IgG (1:2000) for 2 h. The loading control was HRP-conjugated anti-β-actin (1:1500) and did not require a secondary antibody. Bands were visualised by ECL and quantified with ImageJ.

### 2.12 Reverse transcription–quantitative PCR (RT-qPCR)

Total RNA was extracted from testicular tissue and reverse-transcribed into cDNA. Quantitative PCR was performed with 2× Universal Blue SYBR Green Master Mix, with technical triplicates for each sample. Cycling conditions were 95 °C for 30 s, followed by 40 cycles of 95 °C for 15 s and 60 °C for 30 s, and a final melt-curve analysis. GAPDH was used as the reference gene, and relative expression was calculated by the 2−ΔΔCT method. Primer sequences are listed in Table 1.

**TABLE 1:** Primer sequences used for RT-qPCR.

Gene	Upstream primer sequence (5′–3′)	Downstream primer sequence (5′–3′)
GAPDH	CTG​GAG​AAA​CCT​GCC​AAG​TAT​G	GGT​GGA​AGA​ATG​GGA​GTT​GCT
MBOAT2	GCG​GCA​ACC​CAA​ATA​ACA​A	GAG​CCA​GTG​TCT​TGG​TTC​TGA​TTG

### 2.13 Immunohistochemistry

Paraffin sections of testis were dewaxed, rehydrated and subjected to antigen retrieval, incubated overnight at 4 °C with rabbit anti-MBOAT2 primary antibody, then incubated with goat anti-rabbit secondary antibody and developed with DAB. Sections were dehydrated, cleared in xylene, mounted and examined under a light microscope.

### 2.14 Molecular docking

To evaluate potential interactions of AS-IV with AR and MBOAT2, molecular docking was performed for both proteins. The crystal structure of AR (PDB ID: 3G0W) and the AlphaFold model of rat MBOAT2 (AF-Q3T1J2-F1) were obtained from public databases, together with the AS-IV ligand structure (PubChem CID: 13943297). After removal of water molecules and non-target ligands in PyMOL 2.5.2, docking was performed with AutoDock Vina 1.2.

### 2.15 Molecular dynamics simulation

Molecular dynamics simulations were performed with GROMACS 2022. The system was placed in a TIP3P water box, 0.15 mol/L NaCl was added and the charge was neutralised. After energy minimisation, a 100-ns production simulation was run at 310 K and 1 bar. Trajectory analyses included root-mean-square deviation (RMSD), root-mean-square fluctuation (RMSF), radius of gyration (Rg), solvent-accessible surface area (SASA) and the free-energy landscape. Binding free energy was calculated with gmx_MMPBSA.

### 2.16 CCK-8 assay

TM4 cells were seeded in 96-well plates and treated as described in Section 2.4. At the designated time, the medium was replaced with 100 μL fresh medium, 10 μL CCK-8 was added to each well, plates were incubated at 37 °C for 2 h, and absorbance was read at 450 nm with a microplate reader.

### 2.17 C11-BODIPY flow cytometry

After treatment as described in Section 2.4, cells were collected, washed twice with PBS and incubated with probe diluted 1:1000 in assay buffer (0.5 mL per tube) at 37 °C for 15 min. Cells were washed twice more with PBS, resuspended in 500 μL PBS and analysed on a NovoCyte 2060R cytometer.

### 2.18 Statistical analysis

Data are presented as mean ± standard deviation and were analysed with GraphPad Prism 9.0. The Shapiro–Wilk test and Levene’s test were used to assess normality and homogeneity of variance, respectively. Comparisons among groups were performed by one-way analysis of variance followed by Tukey’s *post hoc* test. P < 0.05 was considered statistically significant. For *in vivo* experiments the statistical unit was the animal; for *in vitro* experiments the statistical unit was the independent culture batch.

## 3 Results

### 3.1 AS-IV attenuates pathological testicular injury in VC model rats in a dose-related manner

The molecular formula and chemical structure of AS-IV are shown in Figure 1a. A VC rat model was established by partial ligation of the left renal vein using the Turner method. Rats received vehicle or different doses of AS-IV by daily gavage for 4 weeks (Figure 1b).

During the experiment, the general condition of rats in all groups remained acceptable, body weight increased continuously, and terminal body weight did not differ significantly among groups. No obvious adverse reactions were observed. These results indicate that AS-IV was well tolerated within the dose range tested.

Compared with the blank control group, testicular and epididymal organ indices decreased in the model group; organ indices in the AS-IV groups tended to recover with increasing dose (Figure 1c).

H&E staining (Figure 1e) showed that seminiferous tubules in the blank control and sham-operated groups were relatively intact, germ cells were arranged relatively regularly, and numerous late germ cells or sperm-like structures were present in the lumen. The VC model group showed tubular atrophy, disordered arrangement of germ cells, vacuolation, sloughing of germ cells, fewer sperm-like structures in the lumen and interstitial oedema. These histological changes were lessened after AS-IV intervention and showed a dose-related trend. Testicular morphology in the high-dose group was markedly improved compared with the model group.

Johnsen scores in the blank control and sham-operated groups were both higher than 9.0, whereas scores decreased in the model group (*P* < 0.001); scores increased after AS-IV intervention in a dose-related manner (Figure 1d). Collectively, AS-IV effectively improved VC-induced testicular histological injury, and no obvious toxicity was observed.

### 3.2 AS-IV lessens abnormalities of seminiferous tubules and BTB-associated structure in VC model rats

Scanning electron microscopy (Figure 2a) showed that seminiferous tubules in the blank control and sham-operated groups were relatively intact with relatively smooth surfaces. The model group showed collapse of the tubular wall, irregular or discontinuous basement-membrane-like changes, sloughing of germ cells and reduced luminal contents. After AS-IV intervention, tubular architecture was improved compared with the model group and showed a dose-related trend. These results indicate that AS-IV improved VC-induced abnormalities of seminiferous-tubule structure.

**FIGURE 2:**
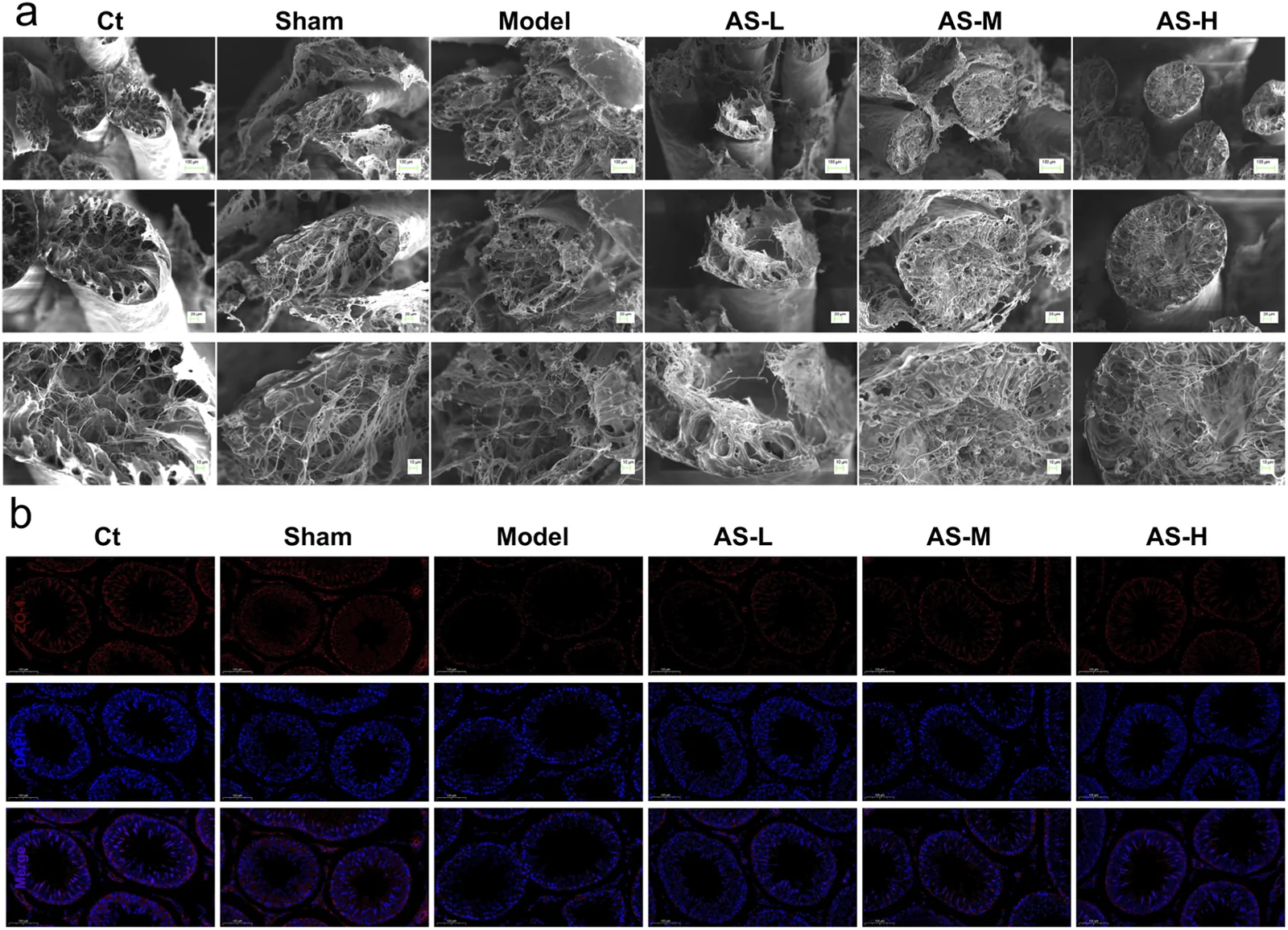
AS-IV restores BTB and seminiferous-tubule structural integrity in VC model rats. **(a)** Representative scanning electron micrographs of seminiferous tubules. Scale bars: as labelled in the images (100, 20 and 10 μm). **(b)** ZO-1 signal in testicular tissue of each group (red, ZO-1; blue, DAPI-stained nuclei). Scale bars: as labelled in figure (100 μm). n = 3 animals.

Immunofluorescence (Figure 2b) showed that ZO-1 signal was located mainly in the basal region of the seminiferous epithelium and in Sertoli tight-junction-associated areas. Signal in the blank control and sham-operated groups was relatively continuous; in the model group ZO-1 signal was weakened and showed a discontinuous distribution; after AS-IV intervention the ZO-1 signal and its continuity were improved. AS-IV restored ZO-1 expression in a clear dose-dependent manner, and the high-dose group approached the sham-operated group.

### 3.3 Proteomics shows that the VC model and AS-IV intervention are associated with changes in lipid metabolism and ferroptosis-related signalling

To explore potential molecular mechanisms of VC testicular injury and AS-IV intervention, label-free quantitative proteomic analysis was performed on testicular tissue from the blank control, VC model and mid-dose AS-IV groups. PCA (Figure 3a) showed clustering within groups and a trend toward separation between groups. Comparison of the blank control and model groups yielded 81 candidate differential proteins, and comparison of the AS-IV and model groups yielded 117 candidate differential proteins; 17 overlapping proteins were shared by the two comparisons (Figure 3b). Heat maps of all 181 candidate differential proteins and of the 17 overlapping proteins are shown in Figures 3c–f. The direction of expression of some proteins in the model group was reversed after AS-IV intervention. Volcano plots showed 28 upregulated and 53 downregulated proteins in the blank control versus model comparison, and 43 upregulated and 74 downregulated proteins in the AS-IV versus model comparison. The screening threshold for candidate differential proteins was |fold change| > 1.2 and *P* < 0.05.

**FIGURE 3:**
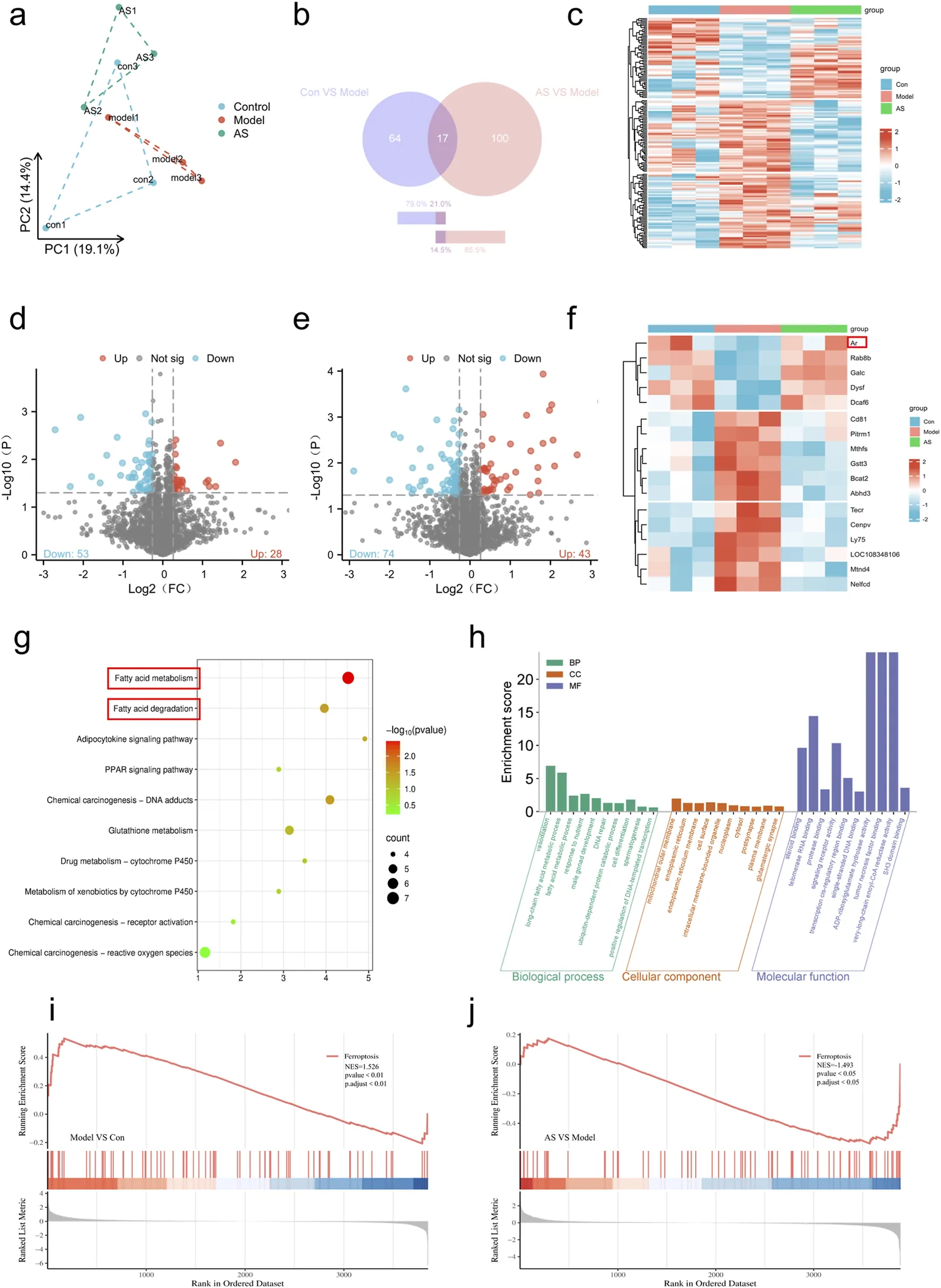
Label-free quantitative proteomic analysis of rat testicular tissue after VC modelling and AS-IV intervention. **(a)** PCA. **(b)** Venn diagram of candidate differential proteins. **(c)** Heat map of all candidate differential proteins. **(d)** Volcano plot of the blank control versus model comparison. **(e)** Volcano plot of the AS-IV versus model comparison. **(f)** Heat map of overlapping candidate proteins. **(g,h)** KEGG and GO enrichment. **(i,j)** GSEA. The screening threshold for candidate differential proteins was |fold change| > 1.2 and nominal *P* < 0.05; n = 3 animals.

KEGG enrichment analysis (Figure 3g) indicated that candidate differential proteins were involved in fatty-acid metabolism and oxidative-stress-related pathways. GO analysis (Figure 3h) showed that biological processes included long-chain fatty-acid metabolism, cellular components included the plasma membrane and mitochondrial membrane, and molecular functions included steroid binding, tumour-necrosis-factor binding and very-long-chain enoyl-CoA reductase activity. The above KEGG and GO enrichment results overlap in part with ferroptosis-related biological processes. Among the 17 overlapping candidate proteins, AR, a recently reported ferroptosis-antagonistic protein, decreased in the model group and increased after AS-IV intervention. These results suggest that ferroptosis-related processes may participate in VC testicular injury.

GSEA of the proteomic data was performed on an online microbioinformatics platform. The ferroptosis gene set was positively enriched in the model group relative to the blank control (NES = 1.526, *P* < 0.01, *P*. adjust <0.01; Figure 3i) and negatively enriched in the AS-IV group relative to the model group (NES = −1.493, *P* < 0.05, *P*. adjust <0.05; Figure 3j). These results provide proteomic evidence for the protective effect of AS-IV.

### 3.4 Fer-1 and AS-IV lessen testicular injury and ferroptosis-related changes in VC model rats

To further evaluate the possible role of ferroptosis in VC testicular injury, a second independent animal experiment was performed, including sham-operated, VC model, low-, mid- and high-dose AS-IV and Fer-1 groups (n = 6 per group; Figure 4a). Terminal body weight did not differ significantly among groups (Figure 4b). The testicular index decreased in the model group (*P* < 0.001) and increased to varying degrees after AS-IV or Fer-1 intervention (Figure 4c). H&E staining and Johnsen scores showed trends consistent with the first cohort. In the Fer-1 group, tubular atrophy, germ-cell sloughing and interstitial oedema were lessened compared with the model group, and the Johnsen score increased (*P* < 0.001; Figures 4d,e). These results confirm that Fer-1 can effectively alleviate VC-induced testicular injury.

**FIGURE 4:**
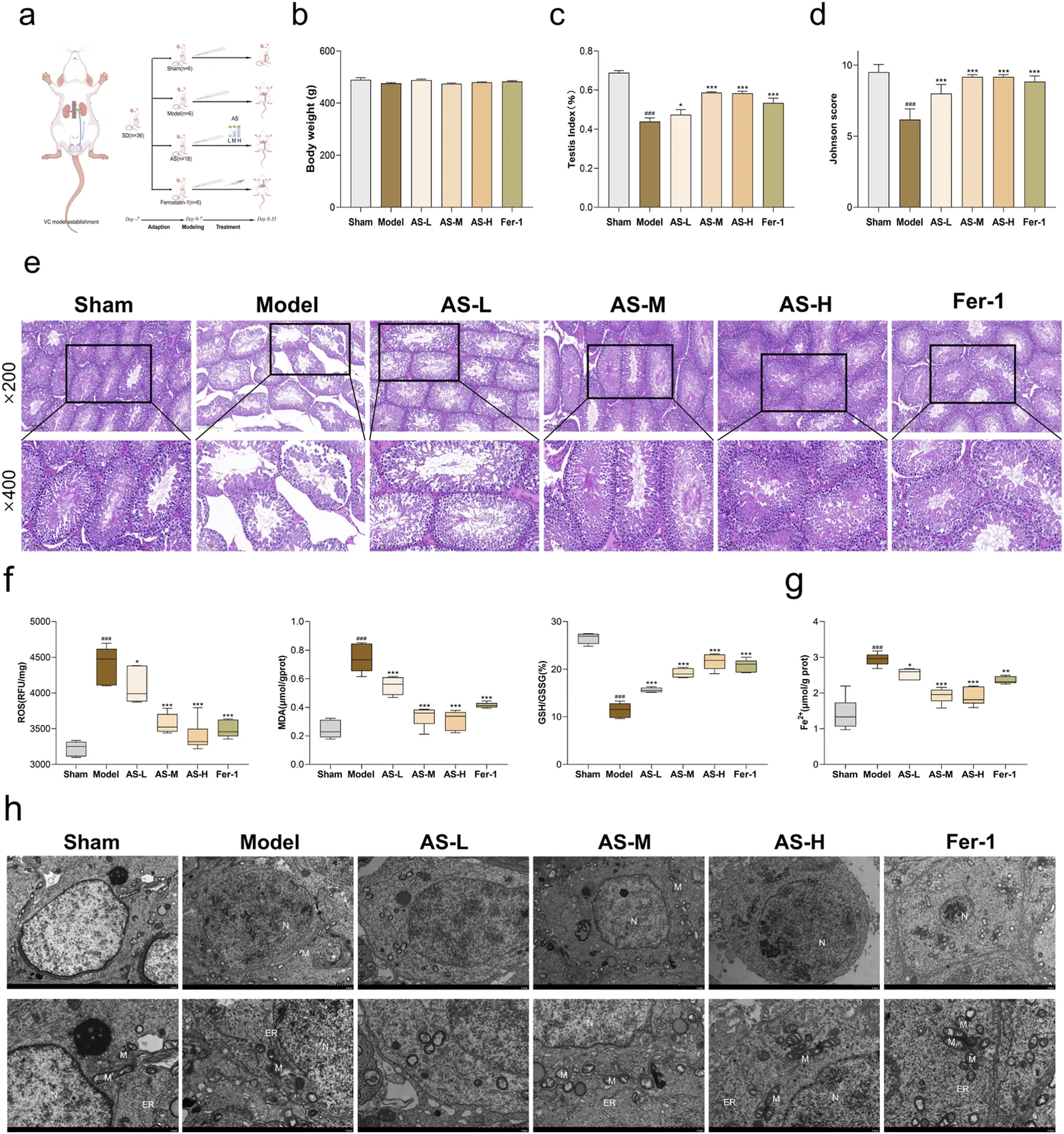
Fer-1 and AS-IV lessen testicular injury and improve ferroptosis-related indices in VC model rats. **(a)** Experimental schedule. **(b)** Terminal body weight. **(c)** Testicular organ index. **(d,e)** Johnsen score and H&E (scale bar, 200 μm). **(f,g)** ROS, MDA, GSH/GSSG and Fe^2+^. **(h)** TEM images (scale bar, 2.0 μm). M, mitochondrion; N, nucleus; ER, endoplasmic reticulum. Bars show mean ± SD. **(b–e)** n = 6; **(f,g)** n = 3; **(h)** representative images. Compared with the sham-operated group: ###*P* < 0.001; compared with the model group: *P < 0.05, **P < 0.01, ***P < 0.001.

Compared with the sham-operated group, ROS and MDA increased and the GSH/GSSG ratio decreased in the VC model group (all *P* < 0.001), and Fe^2+^ content increased (Figure 4f). After AS-IV intervention these indices changed toward the sham-operated group in a dose-related manner. The magnitude of change in the Fer-1 group was broadly similar to that in the mid-dose AS-IV group. High-dose AS-IV restored all indices to levels comparable with the sham-operated group.

TEM (Figure 4h) showed that some mitochondria in the model group were reduced in size, with increased membrane electron density, fewer cristae and vacuole-like changes; these abnormalities were lessened after AS-IV or Fer-1 intervention. These features belong to classical ferroptosis-associated mitochondrial morphology. High-dose AS-IV brought mitochondrial morphology close to normal and afforded protection comparable to that of Fer-1.

Taken together, AS-IV effectively inhibited ferroptosis-related lipid peroxidation and mitochondrial injury in VC testis in a dose-dependent manner, and the Fer-1 results provided further pharmacological support.

### 3.5 Computational prediction of AS-IV interaction with AR/MBOAT2 and tissue expression changes

To explore potential interactions of AS-IV with AR and MBOAT2, molecular docking and 100-ns molecular dynamics simulations were performed. Docking scores were −8.031 kcal/mol for AR and −8.515 kcal/mol for MBOAT2 (Figures 5a,b). In simulations of the AR–AS-IV complex, RMSD tended to stabilise after approximately 20 ns and fluctuated near 1.2 Å; apo-AR tended to stabilise after approximately 60 ns near 1.0 Å. Overall changes in Rg and SASA before and after ligand binding were small, and most RMSF values were below 2.3 Å. In the free-energy landscape, lower-energy conformations were located near an RMSD of approximately 0.11 nm and an Rg of approximately 1.795 nm (Figure 5d). MM/PBSA estimated the AR–AS-IV binding free energy as −7.45 kcal/mol, with relatively large energetic contributions from residues ARG871, LYS912, PRO868, LEU907 and VAL911 (Figure 5e). These computational results suggest potential interactions of AS-IV with AR and MBOAT2 but still require experimental validation.

**FIGURE 5:**
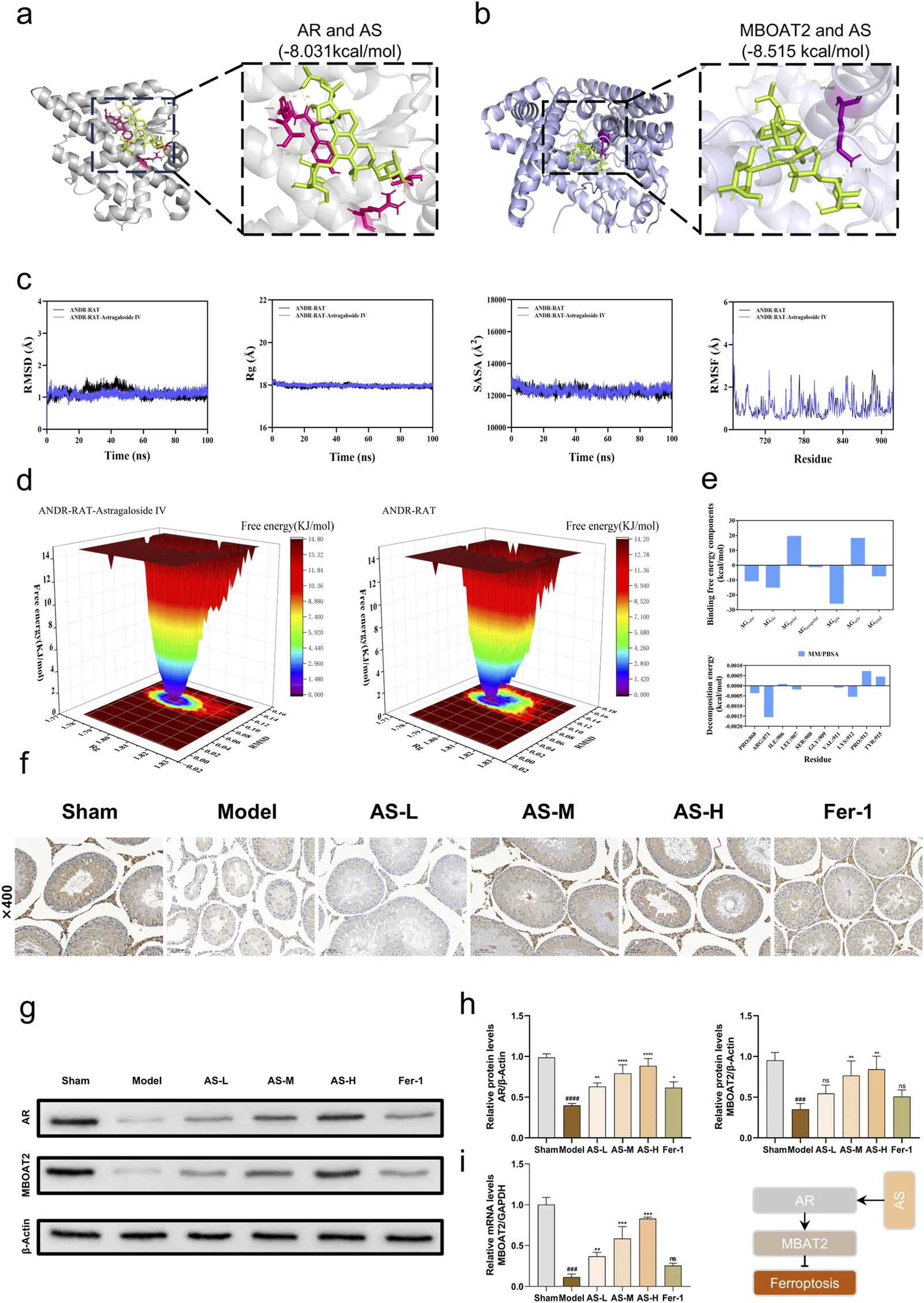
Computational prediction of AS-IV with AR/MBOAT2 and expression changes in testis. **(a)** Molecular docking of AS-IV with AR. **(b)** Molecular docking of AS-IV with MBOAT2. **(c)** RMSD, Rg, SASA and RMSF of the AR–AS-IV complex and of AR. **(d)** Free-energy landscape. **(e)** MM/PBSA estimation. **(f)** MBOAT2 immunohistochemistry; scale bar, 100 μm. **(g,h)** Western blotting of AR and MBOAT2 and quantification. **(i)** Quantification of MBOAT2 mRNA. Bars show mean ± SD; n = 3 animals. Compared with the sham-operated group: ###*P* < 0.001; compared with the model group: *P < 0.05, **P < 0.01, ***P < 0.001.

To observe AR/MBOAT2-related changes *in vivo*, AR and MBOAT2 expression in testicular tissue was examined. Immunohistochemistry showed MBOAT2 signal distributed in seminiferous tubules and interstitial regions. Staining intensity of MBOAT2 decreased in the model group and showed a recovering trend with increasing AS-IV dose (Figure 5f). Western blotting showed that AR and MBOAT2 protein decreased in the model group (*P* < 0.001) and increased with AS-IV dose (Figures 5g,h). AR and MBOAT2 increased only mildly in the Fer-1 group. RT-qPCR showed that MBOAT2 mRNA decreased in the model group (P < 0.001) and increased in a dose-related manner after AS-IV intervention (Figure 5i). These multilevel results indicate that AR/MBOAT2 signalling may participate in the ferroptosis-antagonistic and testis-protective effects of AS-IV.

### 3.6 AS-IV lessens RSL3-induced injury in TM4 cells, and enzalutamide partly weakens this effect

To examine at the cellular level whether AR-related signalling participates in the protective effect of AS-IV, the mouse Sertoli cell line TM4 was used as an *in vitro* pharmacological complement to the rat *in vivo* findings. Ferroptosis-related injury was induced with RSL3 (1.5 μM, last 24 h), and cells were treated with AS-IV (5 μM) and/or enzalutamide (10 μM) for 48 h (Figure 6a).

**FIGURE 6:**
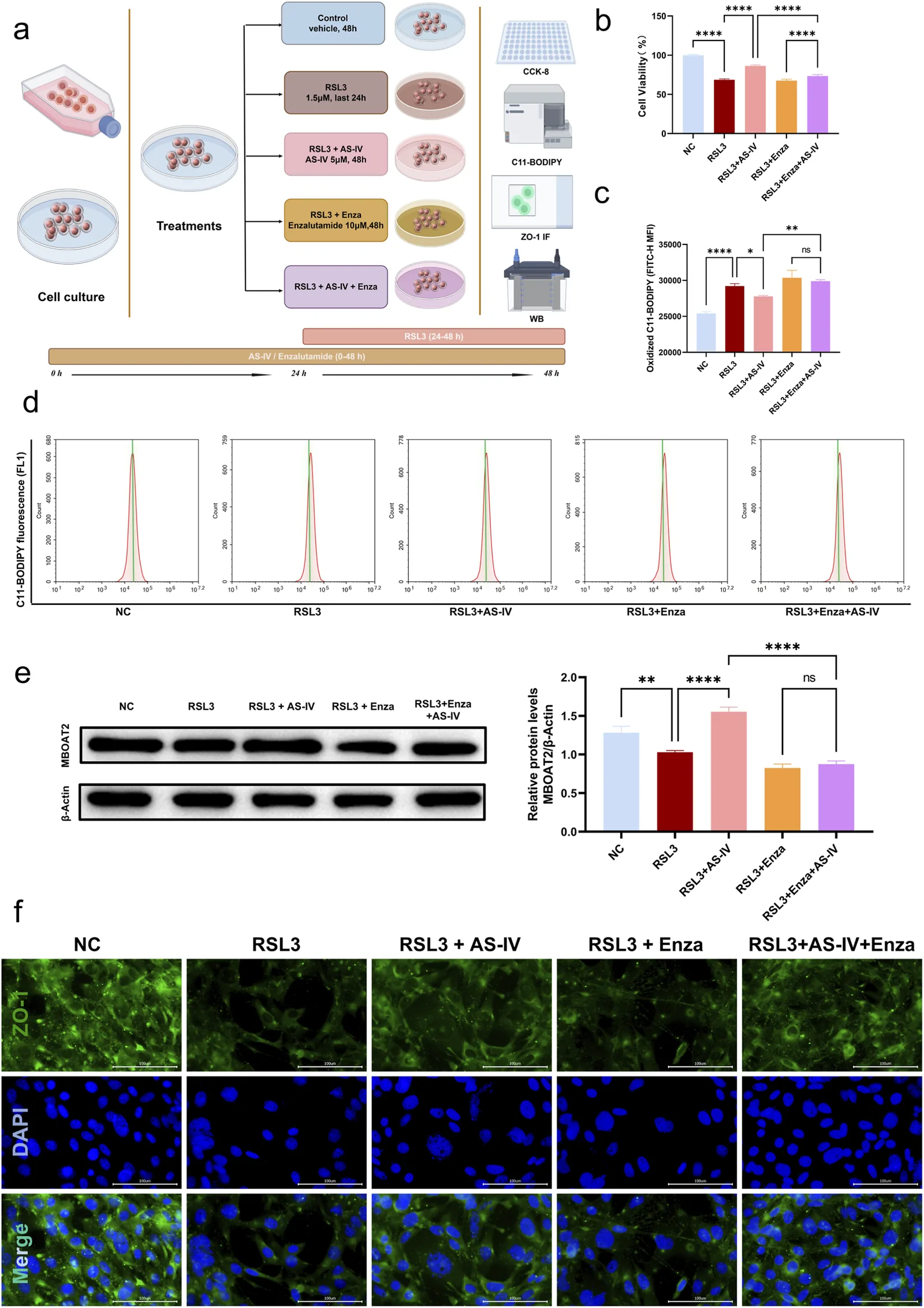
AS-IV lessens RSL3-induced injury in TM4 cells, and enzalutamide partly weakens this effect. **(a)** Experimental schedule. **(b)** CCK-8 cell viability. **(c,d)** FITC-H mean fluorescence intensity of oxidized C11-BODIPY and representative histograms. **(e)** Immunoblotting of MBOAT2 and densitometry relative to β-actin. **(f)** Immunofluorescence of ZO-1 (green) and DAPI (blue). Scale bars, 100 μm. Data are mean ± SD; one-way ANOVA with Tukey’s *post hoc* test. The statistical unit is the independent culture batch (n = 3). *P < 0.05, **P < 0.01, ****P < 0.0001; ns, not significant.

CCK-8 assays showed that, compared with the solvent control, RSL3 significantly reduced cell viability; co-treatment with AS-IV restored viability. Viability in the RSL3 + enzalutamide group was close to that in the RSL3 group; viability in the RSL3 + AS-IV + enzalutamide group remained higher than that in the RSL3 + enzalutamide group but lower than that in the RSL3 + AS-IV group. These findings indicate that enzalutamide partly weakened the improvement of cell viability by AS-IV (Figure 6b).

Live-cell C11-BODIPY 581/591 flow cytometry was used to read membrane lipid peroxidation. Compared with the NC group, RSL3 increased the FITC-H mean fluorescence intensity of the oxidized probe; AS-IV partly reversed this change. In the presence of enzalutamide, AS-IV no longer significantly reduced the oxidized signal, and there was no statistically significant difference between the RSL3 + AS-IV + enzalutamide group and the RSL3 + enzalutamide group (Figures 6c,d).

Immunoblotting showed that MBOAT2 protein after RSL3 treatment was lower than that in the NC control; AS-IV restored MBOAT2. In the presence of enzalutamide, AS-IV failed to restore MBOAT2, and there was no statistically significant difference between the RSL3 + AS-IV + enzalutamide group and the RSL3 + enzalutamide group (Figure 6e). These results suggest that, in this model, upregulation of MBOAT2 protein by AS-IV depends on intact AR pharmacological activity.

Cellular immunofluorescence showed that ZO-1 fluorescence in the NC group was distributed mainly at sites of cell contact. After RSL3 treatment the fluorescence weakened and continuity declined; after AS-IV treatment the fluorescence signal was stronger than in the model group. ZO-1 signal in the RSL3 + AS-IV + enzalutamide group was weaker than in the group given AS-IV alone (Figure 6f).

Taken together, RSL3 can induce loss of viability and lipid peroxidation in TM4 cells, accompanied by decreased MBOAT2 and altered ZO-1 distribution; AS-IV partly reversed these phenotypes. Enzalutamide more completely suppressed the effects of AS-IV on the C11-BODIPY oxidized signal and on MBOAT2 protein, whereas suppression of the viability effect was only partial. These pharmacological results support the involvement of AR/MBOAT2-related signalling in the anti-lipid-peroxidative action of AS-IV, but they are not sufficient to establish AR or MBOAT2 as the sole pathway.

## 4 Discussion

The results of this study show that AS-IV can improve testicular histopathological injury in rats with experimental varicocele. Pharmacologically, AS-IV lessens ferroptosis-related iron accumulation and lipid peroxidation *in vivo* and reduces RSL3-induced lipid peroxidation in TM4 Sertoli cells. Enzalutamide weakens the above protective effects and attenuates the recovery of MBOAT2, suggesting that AR-related signalling participates in the effects of AS-IV.

Varicocele is a common correctable factor contributing to reduced male fertility (Crafa et al., 2024). After impairment of testicular venous return, local hypoxia and oxidative stress appear, and the BTB and seminiferous epithelium are damaged. Persistent oxidative injury interrupts Sertoli cell junctions, reduces germ cells and lowers the Johnsen score (Finelli et al., 2022). Therefore, lessening oxidative stress and protecting the seminiferous-tubule microenvironment are important entry points for improving varicocele-related testicular injury.

Traditional Chinese medicine has shown some promise in the prevention and treatment of male reproductive injury. Qi stagnation with blood stasis, and deficient qi failing to move blood, are common traditional pathogeneses ascribed to varicocele-related testicular injury. Treatments that tonify qi in order to assist blood movement are widely used in the clinic, and Astragali radix is often included in prescriptions for varicocele; AS-IV is its pharmacopoeial quality-control metabolite. Our pharmacological study shows that AS-IV can raise the Johnsen score in rats with experimental varicocele, improve seminiferous-tubule structure and partly restore the tight-junction-associated phenotype reflected by ZO-1, while lowering testicular ROS and MDA and restoring GSH/GSSG. In AS-IV-treated rats the seminiferous epithelium was more orderly and luminal sloughing was reduced. Therefore, AS-IV helps to lessen VC-induced testicular histopathological injury and oxidative stress.

Recent studies have reported that ferroptosis participates in diverse oxidative-stress-related organ injuries (Yuan et al., 2023). However, its role in varicocele-related testicular injury remains unclear. The core feature of ferroptosis is peroxidation of PUFAs in membrane phospholipids, rather than the presence or absence of a single protein; Fe^2+^ accumulation, elevated MDA, GSH depletion and reduced mitochondrial cristae are commonly used as companion readouts (Mishima et al., 2025). Consistent with this, VC rat testes showed increased Fe^2+^, ROS and MDA and a decreased GSH/GSSG ratio, with corresponding mitochondrial changes on TEM; Fer-1 improved these indices. In TM4 cells, RSL3 reduced viability and increased C11-BODIPY oxidation, and AS-IV lessened these changes both *in vivo* and *in vitro*. Fer-1 is not absolutely specific, and confirmatory decreases in total GPX4 and related markers were not observed in the proteome; therefore the present evidence supports the participation of ferroptosis-related lipid peroxidation in VC injury and in AS-IV protection, but does not support regarding it as the sole mechanism.

Lipid homeostasis not only maintains plasma-membrane integrity but also influences stress adaptation (Galliou et al., 2025). Spermatozoa and Sertoli cells are rich in PUFA (Yuan et al., 2023; Li et al., 2024b). Sertoli cells maintain the BTB and the spermatogenic microenvironment through tight junctions (Zhang et al., 2025) and participate in PUFA metabolism (Koeberle et al., 2010). High PUFA content renders these cells more sensitive to lipid peroxidation (Mortensen et al., 2023). After injury to Sertoli membranes, barrier continuity and the luminal microenvironment may both change. This is consistent with the observation in the present study that ZO-1 signal was weakened in VC rats and that AS-IV improved barrier-associated phenotypes.

Recent studies indicate that AR has a ferroptosis-antagonistic action relatively independent of GPX4. Specifically, AR induces transcriptional upregulation of MBOAT2, which remodels polyunsaturated phospholipids into monounsaturated phospholipids and thereby reduces membrane sensitivity to lipid peroxidation (Li et al., 2023). Related to this, AR plays a key role in maintaining seminiferous epithelium and Sertoli cell function (Wang et al., 2022b), and local testicular oxidative stress can suppress AR signalling and affect BTB integrity.

Traditional anti-ferroptotic strategies mainly rely on enhancing GPX4 enzymatic activity, increasing cystine uptake, or interrupting the chain reaction of lipid peroxidation with lipophilic radical-trapping agents such as Fer-1. These interventions act on lipid-peroxidation damage that has already formed and do not necessarily change the PUFA/MUFA composition of membrane phospholipids. By contrast, AR-induced MBOAT2 can reduce peroxidation-prone phospholipids at the substrate level and thereby lower the ferroptosis sensitivity of membranes (Li et al., 2023). We found that AR expression was downregulated in testicular tissue of varicocele model rats, accompanied by decreases in MBOAT2 transcript and protein. After AS-IV intervention, AR protein recovered in part and MBOAT2 transcript and protein were upregulated; enzalutamide weakened the protective effect of AS-IV. These results suggest that AR/MBOAT2-related signalling participates in the process by which AS-IV improves VC testicular injury.

Previous conclusions as to whether VC induces ferroptosis have not been consistent, which may be related to differences in models, detection methods and cell-type specificity. Shaygannia et al. (2021) observed iron accumulation in VC rats but did not find significant changes in the Nrf2–Slc7a11–GPX4 pathway. Studies of human VC semen have reported abnormalities related to ROS, GSH and lipid peroxidation (Sun et al., 2023). Mo et al. (2025) reported upregulation of ACSL4 and proposed that a non-canonical GPX4-related mechanism may exist.

This study was based on the traditional understanding that Astragali radix tonifies qi and moves blood, and evaluated the pharmacopoeial quality-control metabolite AS-IV for protection against experimental varicocele-induced testicular injury. Nevertheless, limitations must be acknowledged. First, knockdown, knockout or overexpression of AR or MBOAT2 was not performed, nor were ChIP, promoter-reporter or target-binding experiments conducted; therefore genetic necessity of this axis cannot be demonstrated, nor can direct binding of AS-IV to AR or MBOAT2 be proved. As an alternative, pharmacological rescue with enzalutamide was performed in TM4 cells. After AR blockade, the effects of AS-IV on lipid peroxidation and MBOAT2 were weakened, whereas the improvement in viability was not completely abolished, thereby supporting the involvement of AR/MBOAT2-related signalling at a pharmacological level. Second, although animal experiments showed improvement in testicular histology and oxidative-stress-related indices, sperm function and mating or pregnancy outcomes were not examined, in contrast to antioxidant studies that used semen parameters in mice (Liu et al., 2025), and the rat VC model differs from clinical varicocele in disease course and compensation; therefore the results cannot be extrapolated to recovery of fertility or to clinical efficacy. Third, phospholipid lipidomics was not performed, and membrane PUFA/MUFA remodelling remains an inference from the literature. Future studies should add transcriptional regulation experiments in testicular cells, genetic intervention of AR and MBOAT2, phospholipid lipidomics, and epididymal sperm and fertility-related endpoints, so that the boundaries of AS-IV action can be defined more accurately.

## 5 Conclusion

This study shows that AS-IV can lessen testicular oxidative stress, lipid-peroxidation-related changes, BTB-associated structural abnormalities and histological injury in rats with experimental varicocele. Pharmacological intervention with Fer-1 and ferroptosis-related indices support the possibility that ferroptosis-related processes participate in VC-induced testicular injury and may also participate in the protective effect of AS-IV. Protection by AS-IV is accompanied by recovery of AR and MBOAT2 expression. In TM4 cells, enzalutamide weakens the improvement of lipid peroxidation and MBOAT2 by AS-IV and only partly weakens the improvement of viability, suggesting that AR/MBOAT2-related signalling is involved but cannot yet be established as a direct target or the sole mechanism. A working model of these findings is shown in Figure 7. These results provide an experimental basis for further study of the role of AS-IV in VC-related testicular injury.

**FIGURE 7:**
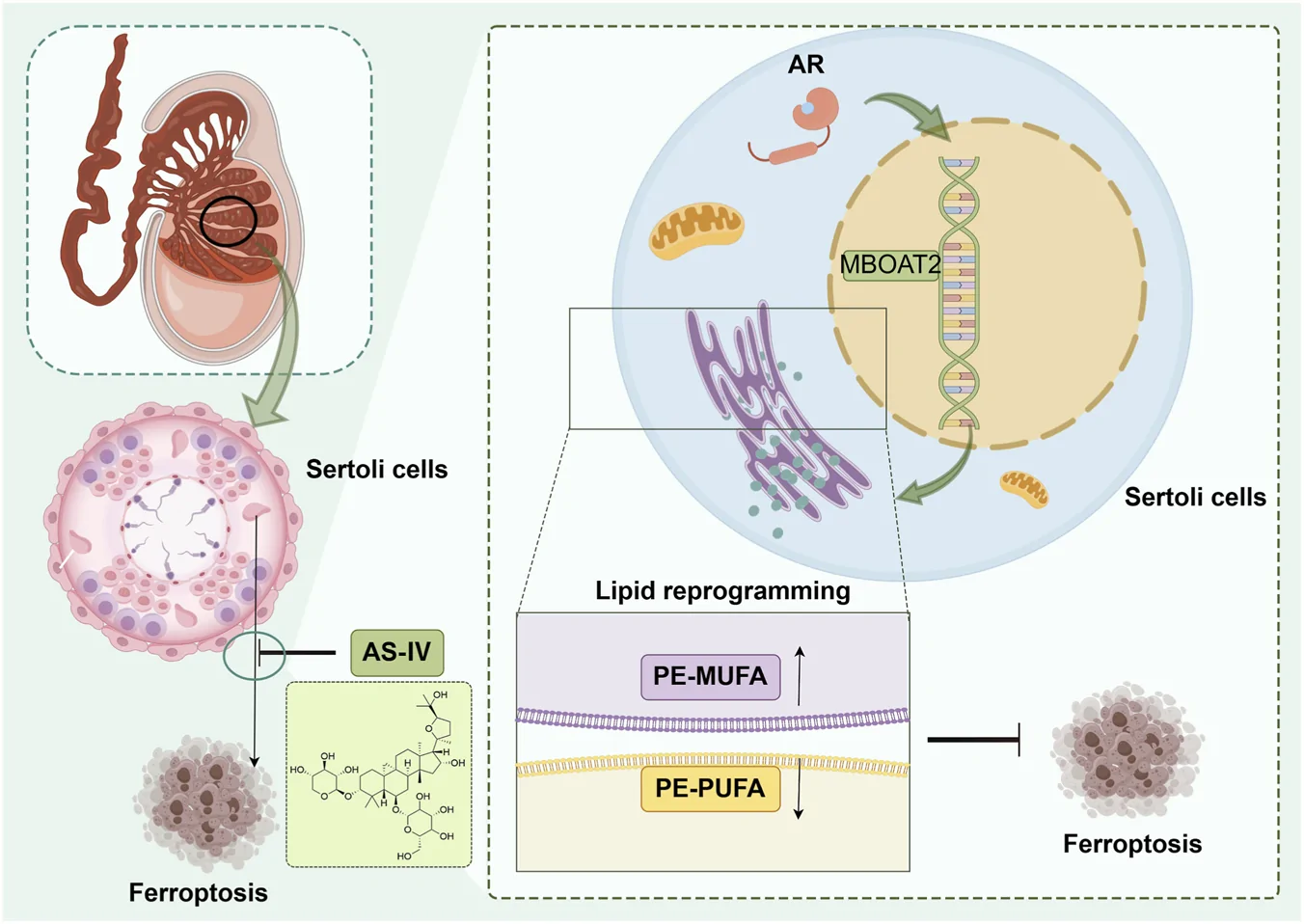
Working model of AS-IV in attenuating experimental varicocele-related testicular injury. The schematic was created with Figdraw (www.figdraw.com).

## Data Availability

The original contributions presented in the study are included in the article. Further inquiries can be directed to the corresponding author.
