# Supplementary material for: Astragaloside IV attenuates ferroptosis-related testicular injury in rats with experimental varicocele
Source: Front Pharmacol. 2026 Oct 7;17:1893309. doi: 10.3389/fphar.2026.1893309 (PMC13683633; doi:10.3389/fphar.2026.1893309)
Supplement: Supplementary file 1 [file Supplementaryfile1.docx]

Supplementary Material

## 1. Supplementary Figures

Supplementary Figure 1

**Supplementary Figure 1.** Proteomic abundance of selected ferroptosis-related proteins in rat testis. (a–h) Relative abundance of SLC7A11, GPX4, ACSL4, FTH1, TFRC, NCOA4, STEAP3 and ATG7 in the Control, Model and AS-IV groups. Bars show mean ± SD (n = 3 biological replicates). These proteins were classified with reference to KEGG pathway rno04216 (Ferroptosis, Rattus norvegicus). The figure is provided to address the request for canonical ferroptosis markers; it does not establish activation or suppression of the GPX4/SLC7A11 axis in this dataset.

**Supplementary Figure 2**

Supplementary Figure 2. Concentration scouting used to select working doses for the TM4 assays. (A) Viability of TM4 cells exposed to enzalutamide (0–20 μM) alone. (B) Viability after RSL3 (0–5 μM) alone. (C) Viability after AS-IV (0–40 μM) alone and after co-treatment with RSL3 (1.5 μM) plus AS-IV (2.5–40 μM). Viability was measured by CCK-8 and is expressed relative to the vehicle control (100%). Bars show mean ± SD from independent culture wells in the scouting run. On the basis of these curves, formal experiments used AS-IV 5 μM and enzalutamide 10 μM for 48 h and RSL3 1.5 μM during the last 24 h, as described in section 2.4 of the main text. These data are dose-finding only and are not used to infer mechanism.

**2 Supplementary Notes**

RSL3 1.5 μM was chosen as a concentration that reduced TM4 viability without collapsing the culture (Supplementary Figure 2B). AS-IV 5 μM was chosen because it did not reduce viability when applied alone and produced a partial rescue of RSL3-induced loss of viability (Supplementary Figure 2C). Enzalutamide 10 μM was chosen as an AR-blocking concentration that left baseline viability largely intact (Supplementary Figure 2A). All working solutions were prepared so that the final solvent concentration was matched across groups.
